# Selected Physical and Mechanical Properties of Particleboards Manufactured from Plantation Willow and Poplar Wood

**DOI:** 10.3390/ma17164069

**Published:** 2024-08-16

**Authors:** Bartłomiej Żabowski, Anita Wronka, Grzegorz Kowaluk

**Affiliations:** 1 Faculty of Wood Technology, Warsaw University of Life Sciences—SGGW, Nowoursynowska St. 159, 02-776 Warsaw, Poland; s207540@sggw.edu.pl; 2 Institute of Wood Science and Furniture, Warsaw University of Life Sciences—SGGW, Nowoursynowska St. 159, 02-776 Warsaw, Poland

**Keywords:** particleboard, willow, poplar, alternative lignocellulosic material, biomass

## Abstract

This research focuses on producing particleboards from the biomass of plantation willow (*Salix viminalis* L.) and poplar (*Populus* spp.), aiming to explore their feasibility as sustainable materials for various applications. Fast-growing willow and poplar are known for their rapid growth and suitability for energy production. They present an intriguing alternative as raw materials with added value for particleboard manufacturing. This study investigates the selected mechanical and physical properties of the produced particleboards, considering parameters such as density profile, bending strength, modulus of elasticity, internal bond, water absorption, thickness swelling, and screw withdrawal resistance. The research results were also compared between different mass shares of willow and poplar particles in the particleboards. The results show that the panels produced entirely from the tested alternative raw materials had a modulus of rupture of 21.7 N mm^−2^ compared to 14.6 N mm^−2^ for the reference panels, with an internal bond of about 2.02 N mm^−2^ compared to 0.65 N mm^−2^ for the reference panels. The thickness swelling after 24 h of soaking was about 24.2% compared to 42.2% for reference panels. The findings underscore the promising potential of willow and poplar-based particleboards as eco-friendly alternatives in the construction and furniture industries, contributing to resource efficiency and carbon emission reduction efforts.

## 1. Introduction

Particleboard is extensively used in furniture production, including desks, countertops, cabinets, flooring panels, wall and ceiling panels, and office partitions. The increasing population is driving a higher demand for these products, leading to a rise in particleboard production [1]. Today’s wood industry faces numerous challenges related to environmental protection, sustainability, and the efficient use of raw materials [2]. Traditional reliance on wood is increasingly problematic in particleboard production due to its diminishing availability and rising costs. This situation compels manufacturers to seek alternative materials with similar or superior properties. Utilizing a raw material in its entirety is not always feasible, as its inherent properties or structure may necessitate alternative applications. This solution is applied to branches considered waste during logging [3,4]. Consequently, a common and straightforward solution is to grind the raw material to an appropriate size and repurpose it for applications such as particleboard production. This approach not only maximizes the use of available resources but also contributes to the efficiency and sustainability of the manufacturing process. Many examples of alternative raw materials for particleboard manufacture may be found in the literature [5,6,7,8]. A literature review indicates significant potential for alder, birch, and larch to be used in the middle layer of particleboard. However, further research is needed to determine how these species can effectively substitute conventional raw materials [9]. Particleboard manufactured from kiwi prunings is one such example [10]. This study aimed to assess the feasibility of using kiwi prunings as a raw material for particleboard manufacture. The researchers used different quantities of kiwi pruning particles and industrial wood particles in the core and surface layers of three-layer particleboards, with commercial urea-formaldehyde (UF) adhesive as the binder. The findings revealed that kiwi stalks had longer fiber length, width, and wall thickness than ordinary forest wood and higher solubility, lignin, and ash levels. Increased use of kiwi pruning particles in the core layer has a negative impact on particleboard attributes. However, with up to 50% kiwi trimming particles in the core, the modulus of rupture (MOR) met the European minimal guidelines for general use. Another study focused on applying saurian (*Toona ciliata* M. Roem) branches and twigs as raw materials for composite particleboards to repurpose saurian stem wood waste. Particleboard and oriented strand board (OSB) were bonded using 10% phenol formaldehyde (PF) adhesive. This research focused on variations in particleboard density and particleboard type. In the conducted tests, the saurian particleboard met only some of the standards despite the different densities of the particleboards produced. This suggests that saurian wood could potentially serve as an admixture in conventional particleboard, though further extensive research is required [11]. Research conducted in Poland has explored using woody raspberry stems as an alternative raw material. Various proportions of raspberry stems were tested, but the spongy structure of the raspberry core resulted in suboptimal strength parameters. Consequently, it was concluded that particles derived from raspberry stems could only serve as an admixture rather than a primary material [12]. The next research example involved creating particleboards using annual cuttings of black chokeberries. Various proportions of this alternative raw material were tested—specifically 10, 25, 50, and 100 by weight. The study revealed that a high bulk density negatively impacts the strength parameters of the particleboards. Consequently, black chokeberry can only serve as a partial additive in the final product [13]. Research has explored the use of particleboards made from mid-branch particles of date palm (*Phoenix dactylifera* L.), which are impregnated with a UF polymer binder. The findings confirmed the feasibility of producing particleboard from date palm branches. Additionally, the study indicated that production parameters could be adjusted to tailor the particleboard for specific applications [14]. Another example is *Sargassum algae*, which has been a nuisance on the coasts of the Caribbean Sea, West Africa, and northeastern Brazil since 2011. The phenomenon has negative environmental and socioeconomic impacts. A study on the feasibility of using sargassum biomass as a raw material for the production of multilayer medium-density particleboard showed that panels with 30% sargassum particles in the core layer and 70% sugarcane bagasse particles in the face layers, bonded with a castor oil-based polyurethane resin, met the minimum requirements for physical and mechanical properties. Furthermore, a life cycle analysis showed that panels produced in Brazil’s Belém, PA region have a lower environmental impact in four of the seven categories assessed than conventional panels. Sargassum can be used as a raw material [15]. Research conducted in Malaysia also analyzed the feasibility of using cassava plant waste, specifically branches, as raw material for producing bio-composite particleboards. In this study, cassava branches were harvested at various maturity stages (6, 9, 12, and 15 months). The results indicated that particleboards made from 12-month-old cassava branches had the best properties, combining strength and durability. Conversely, particleboards from younger (6 and 9 months) and older (15 months) branches exhibited inferior properties. The study suggests that the maturity of cassava branches significantly affects the quality of the resulting particleboard [16]. Further studies focusing on the physical and mechanical properties of particleboard made from the dry branches of *Araucaria angustifolia* and *Eucalyptus grandis* wood were conducted in Brazil. The results showed that specific mass and moisture content remained consistent across treatments. Particleboards with a mix of materials exhibited higher water absorption, especially with rice husk inclusion, due to high silica content affecting particle adhesion. Particleboards using eucalyptus and bamboo performed well, meeting ANSI standards for rigidity and resistance. Rice husk addition reduced these properties, indicating less favorable performance than wood and bamboo [17]. Studies were also conducted in Iran on particleboards with the addition of orange branches. Four types of boards were produced in the study: (100% aspen wood), (50% aspen wood, 25% citrus, 25% old railroad ties), (50% aspen wood, 50% citrus), and (50% aspen wood, 50% old railroad ties), with two levels of resin content (8% and 12%). The research showed that as the content of orange branches in the boards increased, the mechanical strength of the particleboards also increased [18]. In addition to the above-mentioned raw materials, particleboards were also made from chili pepper stalks [19], vine pruning [20], eggplant stalks [21], Greek fir [22] and apple tree branches [23], Nipah palm [24], rice husk [25], sorghum [26], lychee pruning [27], brown seaweed [28] and waste tea leaves [29].

Plantation willow (*Salix viminalis* L.) is a fast-growing plant ideal for biomass production [30]. Its ability to regrow quickly after cutting allows for multiple harvests throughout the year. Additionally, this willow is easy to cultivate and does not require intensive care, making it economically attractive. Similarly, plantation poplar is a fast-growing tree well-suited for short-rotation cropping, allowing for harvests every few years. Poplar’s resistance to various climatic and soil conditions makes it a versatile biomass resource [31,32]. Plantation willow is also valued in land reclamation and environmental protection, particularly in phytoremediation processes, where it helps clean soil and water contaminants [33].

On the other hand, plantation poplar (*Populus* spp.) is used for soil stabilization and air quality improvement due to its carbon dioxide absorption capability [34]. Additionally, plantation willow is used to produce baskets and wicker furniture, while poplar wood, with its low lignin content, is ideal for paper and pulp production in the paper industry. Thanks to their versatile applications and beneficial properties, both plants play an essential role in sustainable development and environmental protection, offering an alternative to traditional wood resources.

Given the rapid growth rate of these alternative raw materials, the concept of integrating them into wood-composite technology emerged. The research aimed to determine the optimal proportion of alternative raw materials, such as plantation willow and poplar, for three-layer particleboard technology.

## 2. Materials and Methods

### 2.1. Materials

The plantation willow was collected in the Lublin Voivodeship. The entire 2–3-year-old shrubs were processed into particles and refined into particles in laboratory conditions without debarking. The initial moisture content was 13%, with a final moisture content after drying of about 3%. The plantation poplar was collected in the Warmian-Masurian Voivodeship. The 5-year-old poplar trees were processed and refined into particles in laboratory conditions. The initial moisture content was 195% because these were freshly cut trees. Like the willow particles, the material was dried to a moisture content of about 3%. The particle fractions used in the particleboards ranged from below 8 mm to 2 mm for the core layer and below 2 mm to 0.25 mm for the face layers. The bark mass content for willow and poplar chips was measured during raw material processing. However, the results showed no significant difference between the raw materials in that field. The bark content for willow was 18.7%, whereas for poplar, it was 19.0%. The bulk density of the produced particles has also been measured [35]. The results were as follows: industrial particles 160 and 157 kg m^−3^, willow 152 and 151 kg m^−3^, and poplar 146 and 148 kg m^−3^, respectively, for face and core layer particles. Industrial UF resin Silekol S-123 (Silekol Sp. z o.o., Kędzierzyn-Koźle, Poland) with a dry matter content of approximately 65%, pH 9.6, and viscosity of 470 mPa·s was used. An aqueous ammonium nitrate solution was used as a hardener to achieve a curing time of the adhesive mass of approximately 85 s at a temperature of 100 °C. No hydrophobic agent was added.

### 2.2. Preparation of Panels

**Figure 1:**
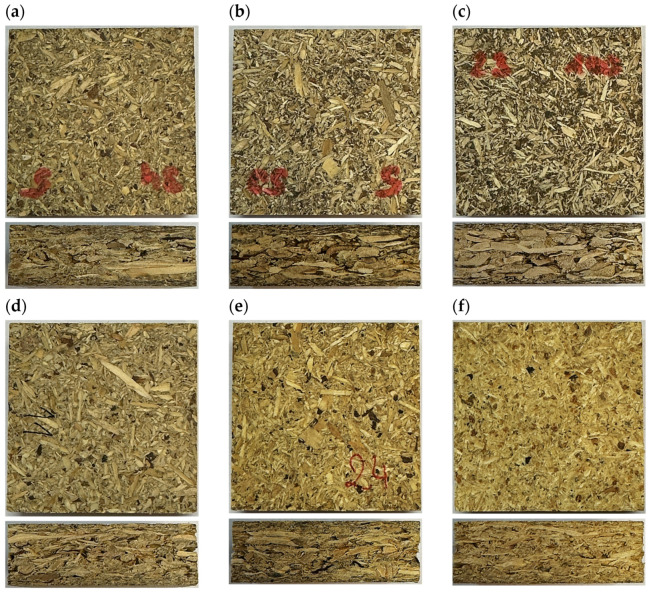
The pictures of selected produced panels (wide-above and narrow-below surfaces): (**a**) W5, (**b**) W50, (**c**) W100, (**d**) P5, (**e**) P50 and (**f**) P100; samples dimensions 50 mm × 50 mm × 16 mm.

Three-layer particleboard with a nominal density of 680 kg m^−3^, dimensions of 320 mm × 320 mm, and thickness of 16 mm were produced. The core (68% of the total panel weight) and the face layer particles were mixed with the adhesive mass separately in a laboratory adhesive drum mixer with the following resination: 12% for the face and 10% for the core layer particles. In the case of fine-grained mixtures and fibers for the face layers, these materials were mixed with the adhesive mass. Pressing was carried out using a hot press (AKE, Mariannelund, Sweden) at parameters close to industrial standards: a pressing temperature of 200 °C, a maximum specific pressure of 2.5 MPa, and a pressing factor of 20 s mm^−1^ of the nominal panel thickness. The mass shares of willow and poplar particles were 5, 10, 25, 50, and 100. Due to this, the produced panels were identified as W5, W10, W25, W50, W100 for willow or P5, P10, P25, P50 and P100, for poplar. The pictures of wide and narrow surfaces of selected produced panels have been depicted in Figure 1. After pressing, the particleboards were calibrated (by sanding). In addition, a reference particleboard was produced according to the same procedure but without adding willow or poplar particles. Before testing, the panels were conditioned at 20 °C and a relative humidity of 65% for seven days.

### 2.3. Characterization of the Elaborated Panels

The following mechanical and physical characteristics were assessed in this study using European standards (where applicable): density [36], bending strength (MOR), and modulus of elasticity (MOE) [37], internal bond (IB) was determined according to EN 319 [38], screw withdrawal resistance (SWR) [39], water absorption (WA) and thickness swelling (TS) after 2 and 24 h of immersion in water [40]. All mechanical properties were tested on a computer-controlled universal testing machine (Research and Development Centre for Wood-Based Panels Sp. z o.o. Czarna Woda, Poland). For every test of mechanical and physical parameters, a minimum of 8 samples of each type of panel were used. For the density profile (DP), the test specimens with dimensions of 50 mm by 50 mm were used, and they were tested using a Grecon DA-X measuring device (Fagus-GreCon Greten GmbH & Co. KG, Alfeld/Hannover, Germany) using direct X-ray densitometry, scanning panel thickness in 0.02 mm increments. After evaluating three samples of each test variant, a representative density profile was chosen for each panel type to be further analyzed. Where possible, the selected results were referenced to the European standard [41].

### 2.4. Statistical Analyses

Analysis of variance (ANOVA) and *t*-test calculations were used to test (α = 0.05) for significant differences between factors and levels, and where appropriate, using IBM SPSS statistic base (IBM, SPSS 20, Armonk, NY, USA). The ANOVA test was followed by Duncan’s test to compare the means. The statistically significant differences achieved are given in Table 1 (non-homogeneous group) whenever the data were evaluated. Where applicable, the mean values of the investigated features and the standard deviation indicated as error bars were presented on the plots.

## 3. Results and Discussion

### 3.1. Water Absorption and Thickness Swelling

**Figure 2:**
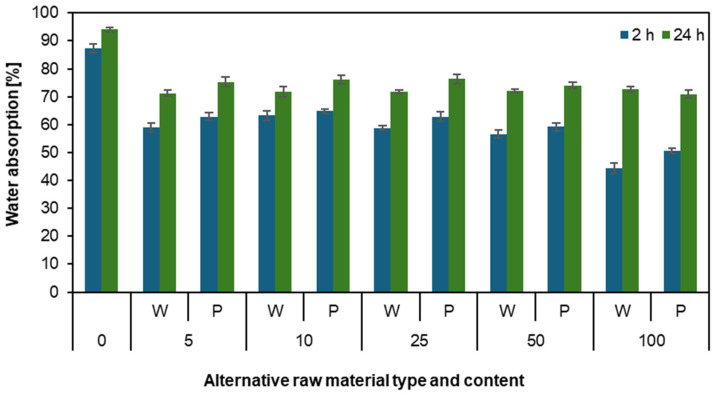
Water absorption of the particleboards produced using various contents of willow and poplar.

Figure 2 represents water absorption after 2 h and 24 h. The results from the first chart show that as the mass parts of willow and poplar increase, the resistance to water absorption also increases. It can also be observed that the charts for willow and poplar are very similar and have comparable results. All results for alternative materials are about 20 percentage points better than the reference particleboard. The lowest water absorption after 2 h is for the particleboard made of 100 willow mass parts (44.3%), and for 24 h, it belongs to the particleboard made of 100 poplar mass parts (70.9%). The particleboard with the highest water absorption is the reference particleboard. These results indicate that particles from willow and poplar are perfectly suitable to produce particleboards, especially for particleboards with increased moisture resistance, such as those used for kitchen countertops or aquarium cabinets. The ability of wood to absorb water depends on several factors, including its anatomy and density. Higher-density wood typically has lower water absorption. Also, the particles with lower bulk density can create a less porous structure that cannot be easily penetrated and impregnated by water. It should be pointed out here that both willow and poplar particles had lower bulk density than industrial particles. In the case of studies conducted on particleboards made with the addition of raspberry particles, an opposite relationship was observed [12]. For both swelling after 2 and 24 h, the greater the mass parts of raspberries in the particleboard, the greater the water absorption. Another example of research conducted on water absorption involved particleboards made from waste plum pits with the addition of hemp fibers. Studies were carried out on boards with 0, 0.5, 0.75, 1, and 1.5 percent shares of hemp fibers. Another example of an alternative raw material is the plant waste of brown mustard (*Brassica juncea* L.). When comparing the water absorption test in this case, there was also a reduced water absorption capacity compared to conventional particleboard [42]. Analyzing the example of single-ply energy willow particleboard with a density of 600 kg m^−3^ and 10% adhesive, a decrease in water absorption (WA) and thickness swelling (TS) was observed, similar to the findings presented in this article [43]. The research showed that as the share of hemp fibers in the particleboard increased, the water absorption of the particleboard also decreased [44].

**Figure 3:**
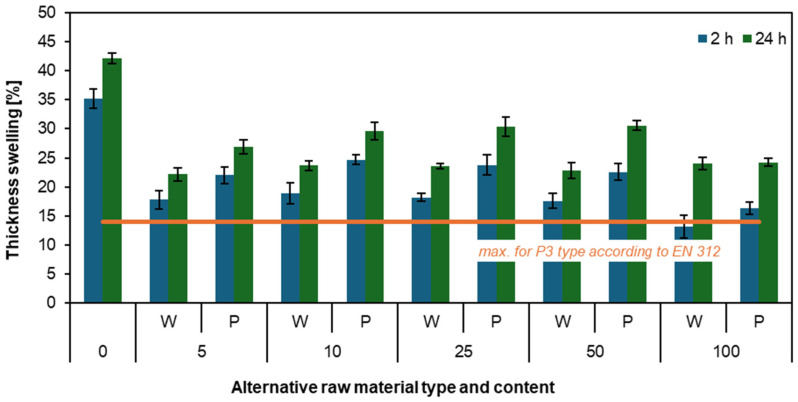
The thickness swelling of the particleboards produced with the use of various contents of willow and poplar particles [41].

Figure 3 describes thickness swelling after 2 h and 24 h. The results are satisfactory due to the close correlation with water absorption results. The higher the mass fraction of willow and poplar, the lower the swelling of the produced particleboards. Compared to the reference particleboard, both willow and poplar particleboards achieved swelling that was even half as much. It should be noted that there are differences between willow and poplar results, with poplar showing higher results by 3 to 5 percentage points for both 2-h and 24-h swelling. The particleboard with 100 willow mass content achieved the best results, with swelling of 13.1% after 2 h and 24.5% after 24 h. It is also worth noting that the 2-h swelling result for this particleboard was the only one that met the EN 312 [41] standard for P3-type particleboards. To clarify, the produced particleboards were intentionally made as P2 type, but swelling results are compared to P3 type standards of EN 312 [41], as there is currently no standard for swelling of P2 type particleboards. In summary, the swelling results from Figure 2 demonstrate that additions of willow and poplar plantation particles increase moisture resistance and significantly reduce swelling of particleboard thickness.

A good comparison of results would be a study on particleboard made from waste beech wood [45]. The study examined particleboards with 10, 20, and 30 parts-by-weight of waste beech wood particles. Similar to willow and poplar, the swelling of particleboards decreased with increasing mass fractions. The particleboard with a 30-parts-by-weight addition of waste beech wood achieved the best results after 2 h, with thickness swelling of 6.7%. In Chile, research was also conducted on *Prunus avium* fruit waste particleboards. The authors noted that in particleboards manufactured from cherry waste and under certain conditions, a slight tendency to increase swelling with an increase in particleboard density could be observed. However, in the context of these studies, no significant differences were found, and the particleboards containing *Prunus avium* waste exhibited less swelling than particleboards made from *Pinus radiata* wood [46].

### 3.2. Determination of Modulus of Rupture and Modulus of Elasticity in Bending and of Bending Strength

**Figure 4:**
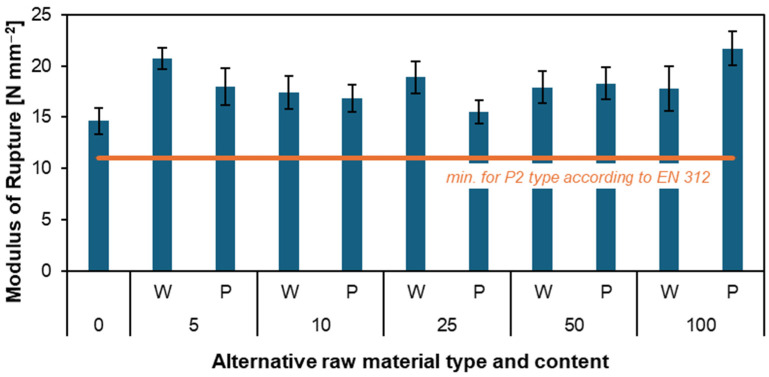
Influence of various content of willow and poplar particles on the MOR of produced particleboard [41].

**Figure 5:**
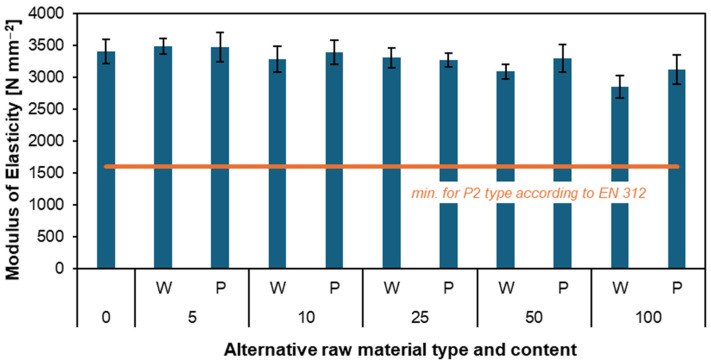
Influence of various willow and poplar particle content on the produced particleboard’s MOE [41].

Figure 4 and Figure 5 present the results of tests on MOR and MOE for produced particleboard. These tests are critical in evaluating the material’s performance. Precisely, the modulus of elasticity measures the material’s stiffness and ability to deform under load. In contrast, the modulus of rupture indicates the maximum stress the material can endure before breaking. In the MOR figure, it is immediately noticeable that, once again, all particleboards made with the addition of willow and poplar have better results than the reference particleboard (14.6 N mm^−2^). For particleboards with added poplar particles, a decrease in results can be observed from 5 (20.7 N mm^−2^) to 25 parts by weight (15.5 N mm^−2^). In subsequent particleboards, MOR results started to increase again up to 100 parts by weight (21.7 N mm^−2^), which, notably, was the particleboard with the highest bending resistance. For the particleboard with added willow particles, we have a similar situation with a decrease in results as the proportion of the alternative material increases: 5 parts by weight (20.7 N mm^−2^) and 10 parts by weight (17.4 N mm^−2^). And an increase of up to 100 parts by weight (17.8 N mm^−2^). The only exception is the W25 particleboard, whose result was as high as 18.9 N mm^−2^. It is also worth noting that all MOR results for the tested samples meet the minimum EN 312 [41] standard.

The MOE graph is interesting because all results, both for the reference particleboard and particleboards with added willow and poplar, are very close. However, it should be noted that with the increasing percentage of willow and poplar particles, the stiffness of the particleboard decreases. The highest MOE results are particleboards W5 (3490 N mm^−2^) and P5 (3476 N mm^−2^). The particleboard with the worst stiffness was W100 (2856 N mm^−2^). Despite the downward trend for increasing mass fractions, all particleboards qualify for the EN 312 standard. Both the MOR and MOE results show that particleboards with added willow and poplar particles are perfectly suitable for industrial production.

Similar studies on MOR and MOE have already been conducted except for single-layer particleboard made from willow [47]. Besides being conducted on single-layer particleboard, these studies also examined the effect of particleboard density (0.57, 0.6, and 0.63 g cm^−3^) and resin content (8, 9, and 10%) on MOR and MOE. The studies showed that as the density and resin content increased, the bending strength and stiffness of the material also increased. The highest results were obtained for a particleboard with a density of 0.63 g cm^−3^ and a resin content of 10%. The MOE was 1610 N mm^−2^, and the MOR was 11.6 N mm^−2^. Compared to our results, these are low values. However, this is due to the absence of the two outer layers in the particleboards. According to Wronka and Kowaluk [12], the lower bulk density of the tested particles can contribute to the higher bending parameters of the investigated particleboards.

In other studies, the modulus of rupture modulus of elasticity for particleboards made from paulownia was also examined. The studies proved that as the particleboard’s density and pressing time increased, the rupture’s modulus also increased. The best results were achieved by particleboard C, with a MOR of 21.5 N mm^−2^ and MOE of 2800 N mm^−2^ [48]. Other studies have shown that adding bamboo layers can significantly raise the MOE and MOR of particleboard. The highest MOE values for the sample with face and back bamboo layers even reached up to 81.64 kg cm^−2^, while the MOR was 670.77 kg cm^−2^ [49].

### 3.3. Screw Withdrawal Resistance

**Figure 6:**
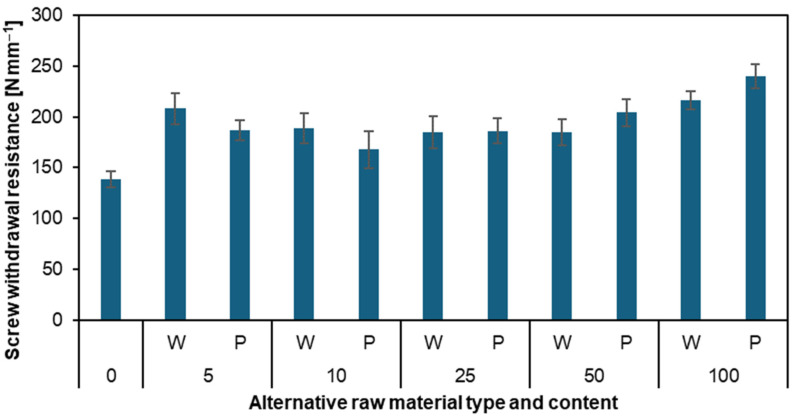
Screw withdrawal resistance of the particleboards produced with the use of various content of willow and poplar particles.

Figure 6 presents the screw withdrawal resistance of the particleboards. Once again, all results for particleboards with added willow and poplar particles were better than those for the reference particleboard (139 N mm^−1^). Similar to the MOR and MOE results, we can observe high screw-holding strength for particleboards W5 (208 N mm^−1^) and P5 (187 N mm^−1^), followed by a decrease in strength for particleboards P10 (168 N mm^−1^) and W25 (185 N mm^−1^). The best results and highest resistance were achieved by particleboards W100 (216 N mm^−1^) and P100 (240 N mm^−1^). Tests on the screw withdrawal resistance of particleboard screws made with black chokeberry particles showed that the addition of chokeberry particles has no significant effect [13]. Research on particleboards and their screw withdrawal resistance was also conducted in Indonesia. The study focused on citric acid-bonded particleboard made from bamboo materials. The research showed that bonding particleboards with citric acid drastically increases their screw withdrawal strength. The average screw withdrawal strength of boards without citric acid was 26.4 N for coarse particles and 36.8 N for fine particles. After adding citric acid, these values increased up to 300 N [50]. This result is higher than that found for boards W100 and P100 in our study.

### 3.4. Internal Bond

**Figure 7:**
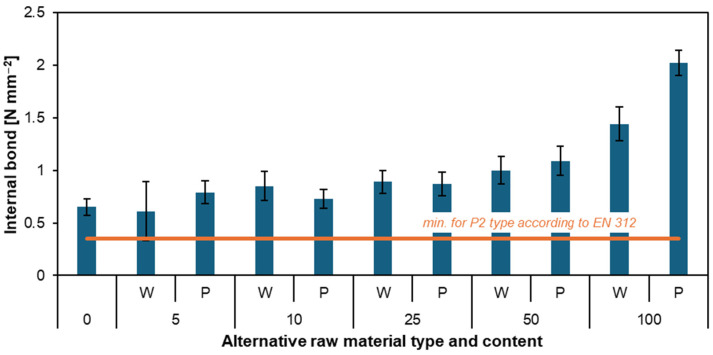
Internal bonds of the particleboards are produced using various willow and poplar particle contents [41].

Figure 7 presents the IB results of the particleboards. This test describes how well the particles in the core layer of the particleboard are bonded together. Once again, the reference particleboard showed the lowest result (0.65 N mm^−2^). It can also be observed that with the increase in the mass proportions of willow and poplar in the particleboard, the IB increases. The differences in IB values between particleboards W5, P5, and particleboards W50, P50 range from 0.39 to 0.65 N mm^−2^. Particleboards W100 (1.44 N mm^−2^) and P100 (2.02 N mm^−2^) achieved the most satisfactory and highest results. These are very high results compared to the rest of the particleboards, especially compared to the EN 312 [41] standard of 0.35 N mm^−2^. Such results could be due to the different cross-sections of willow and poplar particles compared to industrial components. Thanks to this cross-section, the particles in the middle layer of the particleboard could bond better. The studies prove that the addition of willow and poplar particles positively affects the IB perpendicular to the plane of the particleboard.

Polish researchers conducted a study to produce a three-layer particleboard with varying amounts of willow in the core layer. They created three variants: 0, 50, and 100 parts by weight of willow relative to conventional wood particles. The IB tests revealed that the IB strength increases as the proportion of willow in the core layer increases, which aligns with the findings discussed in this article [51]. The lower bulk density of the particles used for particleboard production also significantly influences the IB [12]. Another example involves testing a single-layer willow particleboard to simulate a core layer. The studies focused on the proportion of adhesive used and the density of the board. The results were slightly lower than expected in one variant, with 10% adhesive and a density of approximately 630 kg m^−3^. However, it is essential to note that these particles lacked surface layers, which are crucial in three-layer particleboard parameters. The study confirmed that density influences IB, and the density of the tested particleboards was 670 kg m^−3^, aligning with the observed trend [47]. Studies have already been conducted on particleboards made with different mass proportions of poplar (*Populus alba* L.) [52]. These studies aimed to produce particleboards with varying proportions of mass of poplar particles and sunflower stalks. The next step was to examine their physical and mechanical properties. Particleboards were produced in proportions of 0, 25, 50, 75, and 100 parts based on the weight of alternative raw materials. The results for the internal bond perpendicular to the plane were 0.46, 0.53, 0.47, and 0.69 N mm^−2^, respectively.

In both these studies and the current one, the particleboard with 100 parts by weight poplar content achieved the best results. However, these results are significantly lower compared to the current study. This difference is due to the varying content of sunflower stalk parts, which can negatively impact the tensile strength perpendicular to the plane of the particleboard [52]. Other studies were also conducted regarding particleboards’ IB. The aim of the research was to evaluate *Acacia saligna*, *Conocarpus erectus*, *Melia azedarach*, and date palm (*Phoenix dactylifera*) for their suitability in particleboard production. The studies showed that the values of IB for all species and both density levels met the minimum requirement. The best results were achieved by particleboard from *Conocarpus erectus* with an IB value of 1.02 MPa [53]. This is a comparable result to the W50 particleboard from our study. Internal bonds for particleboards manufactured with walnut wood residues also increased with the proportion of the alternative raw material [54].

### 3.5. Density Profiles

**Figure 8:**
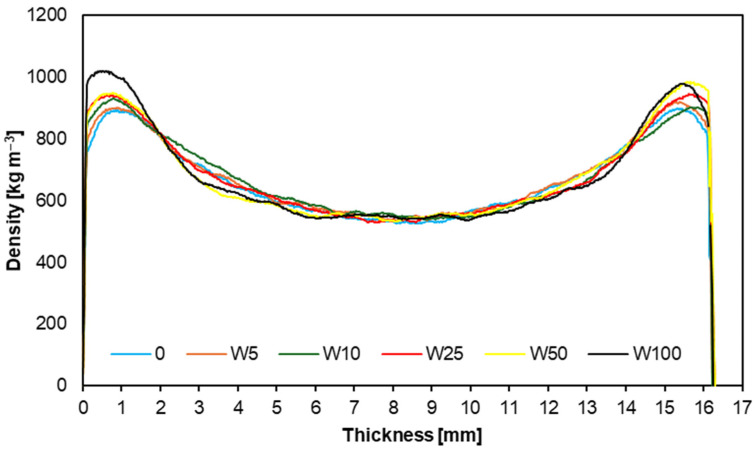
Density profiles of tested samples from willow.

**Figure 9:**
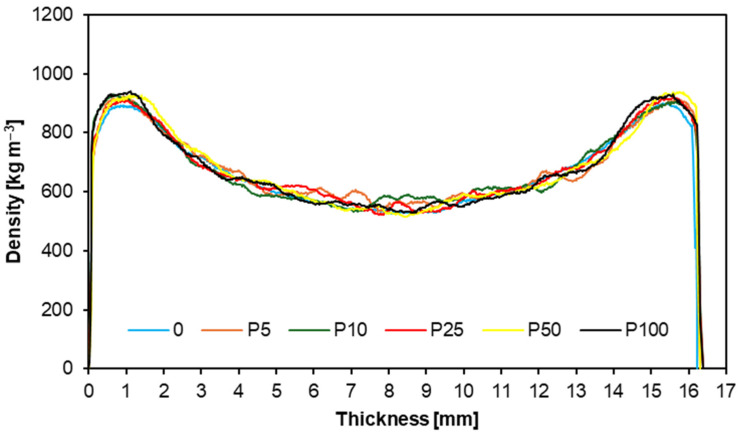
Density profiles of tested samples from poplar.

Figure 8 and Figure 9 illustrate the density profiles of the tested samples, showing how the density of the particleboard changes across its cross-section. The results indicate that the profiles for both willow and poplar overlap, with a smooth density transition from the outer layers to the core layer. The primary difference between the profiles is the variable density in the core layer of the particleboards with the poplar addition. This study also determined the overall particleboard densities: the particleboards with willow ranged from 671 to 680 kg m^−3^, while those with poplar ranged from 671 to 683 kg m^−3^. These results are very satisfactory, as they align with the nominally planned densities for the particleboards. A similar density profile was obtained in the study, where the alternative raw materials were branches from the annual maintenance of plum and apple trees [55].

## 4. Conclusions

This study aimed to determine whether there are differences between particleboards produced with the addition of particles from plantation willow and poplar wood. The second objective was to evaluate the viability of using these unconventional lignocellulosic materials for mass production. The research shows that particles from willow and poplar yield very similar results. This is confirmed by tests such as water absorption, thickness swelling, modulus of rupture, modulus of elasticity, screw withdrawal resistance, internal bond, and density profile. These tests also demonstrate that the alternative raw materials selected are ideally suited for use as additives in industrial particleboard production.

The properties of willow and poplar that had the most significant impact on the results and deserved the most outstanding recognition include reducing water absorption and swelling in the particleboard, increasing screw withdrawal resistance, and significantly enhancing perpendicular internal bonds. It is also worth noting that all particleboards with plantation willow and poplar particles comply with EN standards.

We believe the particleboards produced by the alternative raw materials can successfully compete with the commercial furniture particleboards and, by proper binder selection, could also serve in structural applications, for example, in wooden frame prefabricated buildings. Further investigations into the non-energetic utilization of willow and poplar wood in wood-based composites will probably be directed to optimizing the particle’s shape and dimensions, as well as attempts to step away from formaldehyde-based binders.

## Figures and Tables

**Table 1:** The statistical assessment results of mean values.

Test Type	Alternative Raw Material Particles Share [%]										
	0	5		10		25		50		100	
		W	P	W	P	W	P	W	P	W	P
MOE	a *	a	a	a	a	a	a	b	a	c	b
MOR	a	b	c	c	c	b, c	c	c	c	c	b
IB	a	a	a	a, b	a	b	b	b	b, c	d	e
SWR	a	b	c	c	c	c	c	c	b	b	d
TS 2 h	a	b	c	b	c	b	c	b	c	d	b
TS 24 h	a	b	c	b	c, d	b	d	b	d	b	b
WA 2 h	a	b	c	c	c	d	b, c	e	d	f	g
WA 24 h	a	b	c	b	c	b	c	d	b	d	d

* a–g homogeneous group.

## Data Availability

The data presented in this study are available in the open-access repository: https://doi.org/10.18150/QLCLZF.
